# Age standardization and time-of-day performance for the Oldenburg Sentence Test (OLSA): results from the population-based Gutenberg Health Study

**DOI:** 10.1007/s00405-023-08358-2

**Published:** 2023-12-18

**Authors:** Karoline O’Brien, Berit Hackenberg, Julia Döge, Andrea Bohnert, Tobias Rader, Karl J. Lackner, Manfred E. Beutel, Thomas Münzel, Philipp S. Wild, Julian Chalabi, Alexander K. Schuster, Irene Schmidtmann, Christoph Matthias, Katharina Bahr-Hamm

**Affiliations:** 1 Department of Otorhinolaryngology, University Medical Center Mainz, Langenbeckstrasse 1, 55131 Mainz, Germany; 2 Institute for Clinical Chemistry and Laboratory Medicine, University Medical Center Mainz, Mainz, Germany; 3 Department of Psychosomatic Medicine and Psychotherapy, University Medical Center Mainz, Mainz, Germany; 4 Department of Cardiology-Cardiology I, University Medical Center Mainz, Mainz, Germany; 5 Department of Ophthalmology, University Medical Center Mainz, Mainz, Germany; 6 Preventive Cardiology and Preventive Medicine, Department of Cardiology, University Medical Center Mainz, Mainz, Germany; 7 Institute of Medical Biostatistics, Epidemiology and Informatics, University Medical Center Mainz, Mainz, Germany; 8 Center for Thrombosis and Hemostasis (CTH), University Medical Center Mainz, Mainz, Germany; 9 German Center for Cardiovascular Research (DZHK), Partner Site RhineMine, Mainz, Germany; 10 Division of Audiology, Department of Otolaryngology, University Hospital, Ludwig-Maximillian-University, Munich, Germany

**Keywords:** German Matrix Test [Oldenburg Sentence test (OLSA)], Hearing loss, Speech intelligibility, Age standardization, Speech audiometry

## Abstract

**Purpose:**

The Oldenburg Sentence Test (OLSA) is a German matrix test designed to determine speech recognition thresholds (SRT). It is widely used for hearing-aids and cochlear implant fitting, but an age-adjusted standard is still lacking. In addition, knowing that the ability to concentrate is an important factor in OLSA performance, we hypothesized that OLSA performance would depend on the time of day it was administered. The aim of this study was to propose an age standardization for the OLSA and to determine its diurnal performance.

**Methods:**

The Gutenberg Health Study is an ongoing population-based study and designed as a single-centre observational, prospective cohort study. Participants were interviewed about common otologic symptoms and tested with pure-tone audiometry and OLSA. Two groups—subjects with and without hearing loss—were established. The OLSA was performed in two runs. The SRT was evaluated for each participant. Results were characterized by age in 5-year cohorts, gender and speech recognition threshold (SRT). A time stamp with an hourly interval was also implemented.

**Results:**

The mean OLSA SRT was − 6.9 ± 1.0 dB (group 1 male) and − 7.1 ± 0.8 dB (group 1 female) showing an inverse relationship with age in the whole cohort, whereas a linear increase was observed in those without hearing loss. OLSA-SRT values increased more in males than in females with increasing age. No statistical significance was found for the diurnal performance.

**Conclusions:**

A study with 2900 evaluable Oldenburg Sentence Tests is a novelty and representative for the population of Mainz and its surroundings. We postulate an age- and gender-standardized scale for the evaluation of the OLSA. In fact, with an intergroup standard deviation (of about 1.5 dB) compared to the age dependence of 0.7 dB/10 years, this age normalization should be considered as clinically relevant.

## Introduction

More than 1.5 billion people suffer from hearing loss [1]. With demographic changes and increasing life expectancy, the prevalence of hearing loss is expected to increase steadily [1].

At the level of the individual, hearing loss is an enormous burden on social well-being [2]: the inability to communicate with others inevitably leads to social exclusion and loss of productivity. Because hearing loss usually develops slowly, it often goes unnoticed and patients remain untreated [3]. Age-related hearing loss is a gradual process that progresses almost unnoticed from an individual perspective, but has long been recognized as a major health problem in aging societies [4]. It is one of the most common chronic conditions and the most common sensory deficit in an aging society [5]. Although the fact is well known, presbyacusis is underdiagnosed and undertreated [6]. A systematic review on the prevalence of hearing loss in Germany in 2018 could only identify six studies (ten publications) providing data on the prevalence of hearing impairment in Germany [7]. A recent study found a prevalence of 12.7% for moderate to profound hearing loss [8]. Although a number of large cohort studies reporting on audiometric data have been published, the results are difficult to compare due to different definitions of hearing loss and different testing methods [5].

The onset of hearing loss is often manifested by problems with communication and speech perception at various levels of background noise. Presbycusis develops gradually over time and has a significant impact on daily life. The risk of memory loss is increased [9], as is the risk of accelerated development of dementia [10] and depression [11]. Löhler et al. suggested a representative epidemiologic study considering age-dependent frequency-specific definitions of hearing loss [7].

The German Matrix Test (OLSA) is a test for speech perception in noise with a large number of repeatable test lists [12]. It is not only commonly used to measure speech intelligibility in noise, but is also effective for cochlear implant (CI) listening tests [13].

There is currently no official age standardization of the OLSA for adults. In contrast, the Oldenburger Kindersprachtest (OLKISA) is age-standardized to allow for scoring adjustments. The OLKISA can be administered to children 4 years of age and older [14] and is child-friendly due to test lists of shortened sentences. The application of the OLKISA in clinical practice is also extended to adults with a reduced word span, as well as to adults with cochlear implants (if the OLSA cannot be performed) or in adults with a short concentration span.

The purpose of this paper is to assess the dependence of the German Matrix Test on age. Considering the increasing hearing loss in an aging population and eliminating this factor, we assume that it is possible to postulate an age standardization for the German Matrix Test.

In addition to the lack of age categorization, there are no data available for the OLSA regarding diurnal performance. The OLSA test procedure requires a certain level of attention, concentration, and cognitive fitness on the part of the subject. In addition, OLSA results are also dependent on auditory working memory [15]. It might be expected that testing later in a day that includes a full schedule of tests and examinations, some of which require concentration, would lead to poorer results.

To address this lack of data, the purpose of this study is to report on the time-of-day dependency of the OLSA, to further understand the possibility of an age dependency and to establish an age and possibly gender categorization for this test.

Until now, a large population-based study for the German Matrix Test has been lacking. This study aims to change that by reporting on the age distribution in men and women as well as the time-of-day dependency in a population-based, randomly selected cohort study. This is the largest study evaluating OLSA data in Germany known to the authors.

## Methods

The Gutenberg Health Study (GHS) is a large, ongoing population-based study, designed as a single-centre, observational, prospective cohort study. It was initiated in 2007 at the University Hospital of Mainz, Germany, and is planned to cover the population of the city of Mainz and its district of Mainz-Bingen, Germany. It was approved by the Institutional Review Board (Ethics Commission of Rhineland-Palatine, reference no. 837.020.07). Written informed consent, in accordance with the Declaration of Helsinki, was obtained from all participants before participation in the study. The population sample was randomly selected from the civil registry and stratified by age, sex and residence (rural vs. urban). Physical and mental disabilities that might prevent the participant from attending the study site were an exclusion factor. Insufficient knowledge of the German language was also an exclusion criterion. In 2017, (10-year follow-up) additional otological examinations were included in the study. A full description of the study design has been published previously [16].

All examinations of the participants took place on the premises of the University Hospital Mainz. The study nurses were trained and continuously educated by certified audiology assistants from the Department of Otolaryngology and Audiology at the University Hospital Mainz. The implementation of a standard operating procedure (SOP) ensured the validity of the audiological examinations. The ENT evaluation, and therefore the OLSA, was performed at different times of the day, the earliest at 10:00 am and the latest being 8:00 pm.

After an interview about common otologic symptoms (i.e., tinnitus), pure-tone audiometry for air- and bone-conduction was performed separately for both ears at the following frequencies: 0.125, 0.25, 0.5, 0.75, 1, 2, 3, 4, 6, 8, and 10 kHz. All tests were performed with an Auritec AT1000 clinical audiometer and in a soundproof booth. The adaptive procedure of the commercially available German Matrix Test (OLSA) was used as described by Brand et al. 2002 in an open version [17]. The software for the German Matrix Test is called “Oldenburger Messprogramme” by Hörtech R&D.

Before the speech audiometry, an otoscopy (observation of the external auditory canal and the tympanic membrane) was performed to rule out any impairment of the auditory canal.

In addition, the OLSA was performed in two consecutive runs (trial and test, each with 20 sentences). The SRT was documented for each participant for both runs. The OLSA consists of five words (name—verb—number—adjective—object) with a possible combination of 50 words. It is a randomized, adaptive procedure with a fixed noise level to a varying speech level or a varying speech level to a fixed noise level. The noise signal was generated by summing and averaging the time signals of a large number of OLSA test sentences (long-term speech spectrum). Participants with missing data at 0.5, 1, 2 or 4 kHz were excluded from the study, as were those with missing data for OLSA.

### Statistical analyses

Descriptive statistics were computed separately for age intervals (5-year intervals), the time-of-day testing, sex and the OLSA speech recognition threshold (SRT).

Participants were divided into groups according to their age. Group 1: 25–29 years of age (y), group 2: 30–34 y, group 3: 35–39 y, group 4: 40–44 y, group 5: 45–49 y, group 6: 50–54 y, group 7: 55–59 y, group 8: 60–64 y, group 9: 65–69 y, group 10: 70–74 y, group 11: 75–79 y, group 12: 80–84 y, group 13 85–89 y. Each age group was subdivided by sex. Means and standard deviations were reported. Analysis of Variance (ANOVA) was performed to test the contribution of hearing loss and age to the SRT. A subcohort including only individuals without hearing loss (mean hearing loss < 20 dB at frequencies 0.5, 1, 2 and 4 kHz according to WHO) was created and analyzed separately in order to exclude the effect of hearing loss. Levene's test was used to test homogeneity of variance, and Dunnett's test was used to compare differences in variances with respect to the youngest age decades as a control group.

Furthermore, a time stamp with an hourly interval (i.e. 10:00 am–11:00 am, 11:00 am–12:00 pm etc.) was implemented and the measurements at these time points were compared.

All statistical analyses were performed using R version 3.6.1 (2019-07-05) and gnuplot (5.4.2) software for graphical design.

## Results

**Fig. 1:**
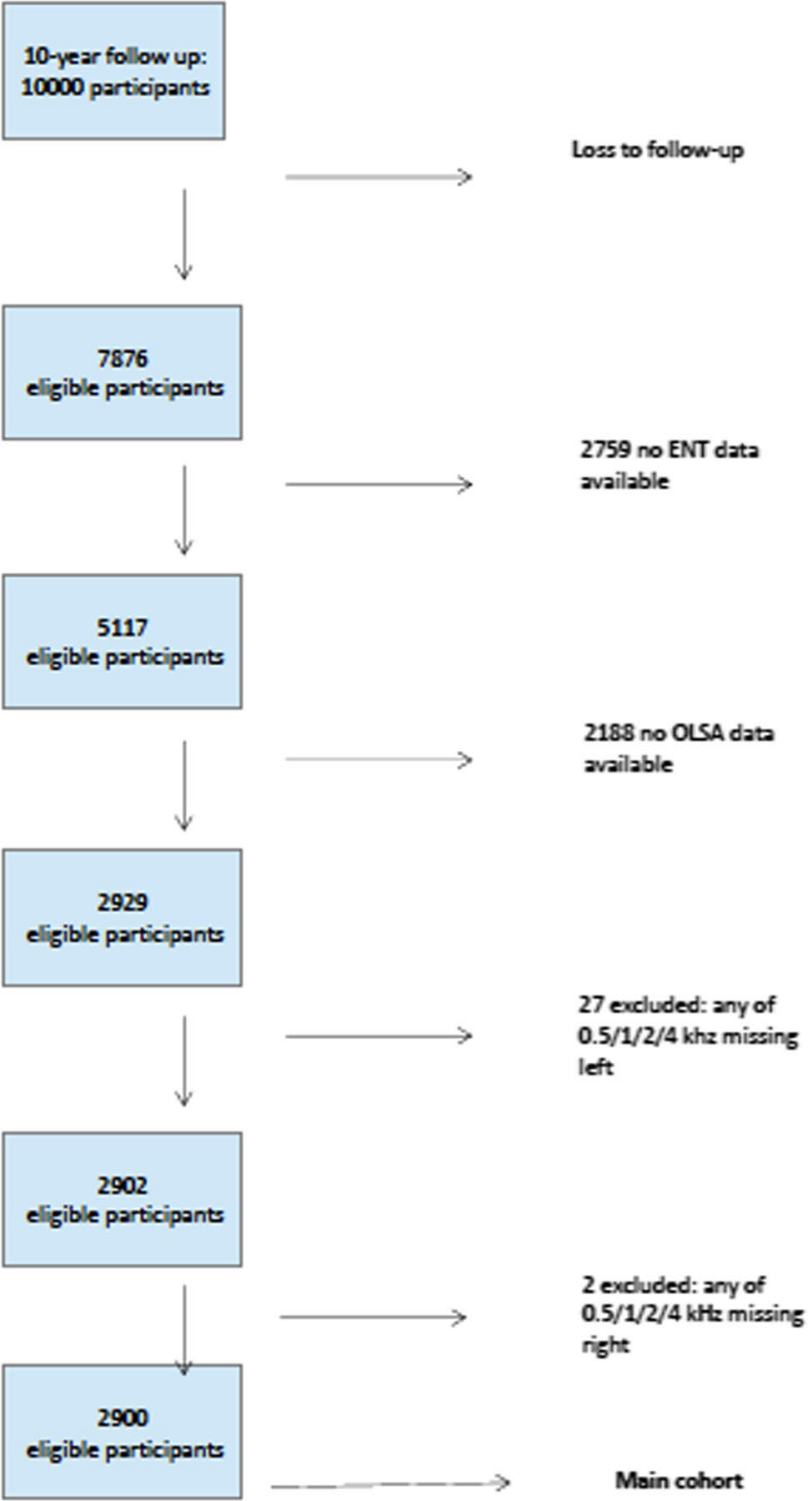
Flow chart of participant selection

In the Gutenberg Health Study 10,000, participants were invited to visit the study site for their 10-year follow-up examination. Complete data on OLSA were available for 2900 participants (main cohort), see the flowchart (Fig. 1) for the study group selection process.

**Fig. 2:**
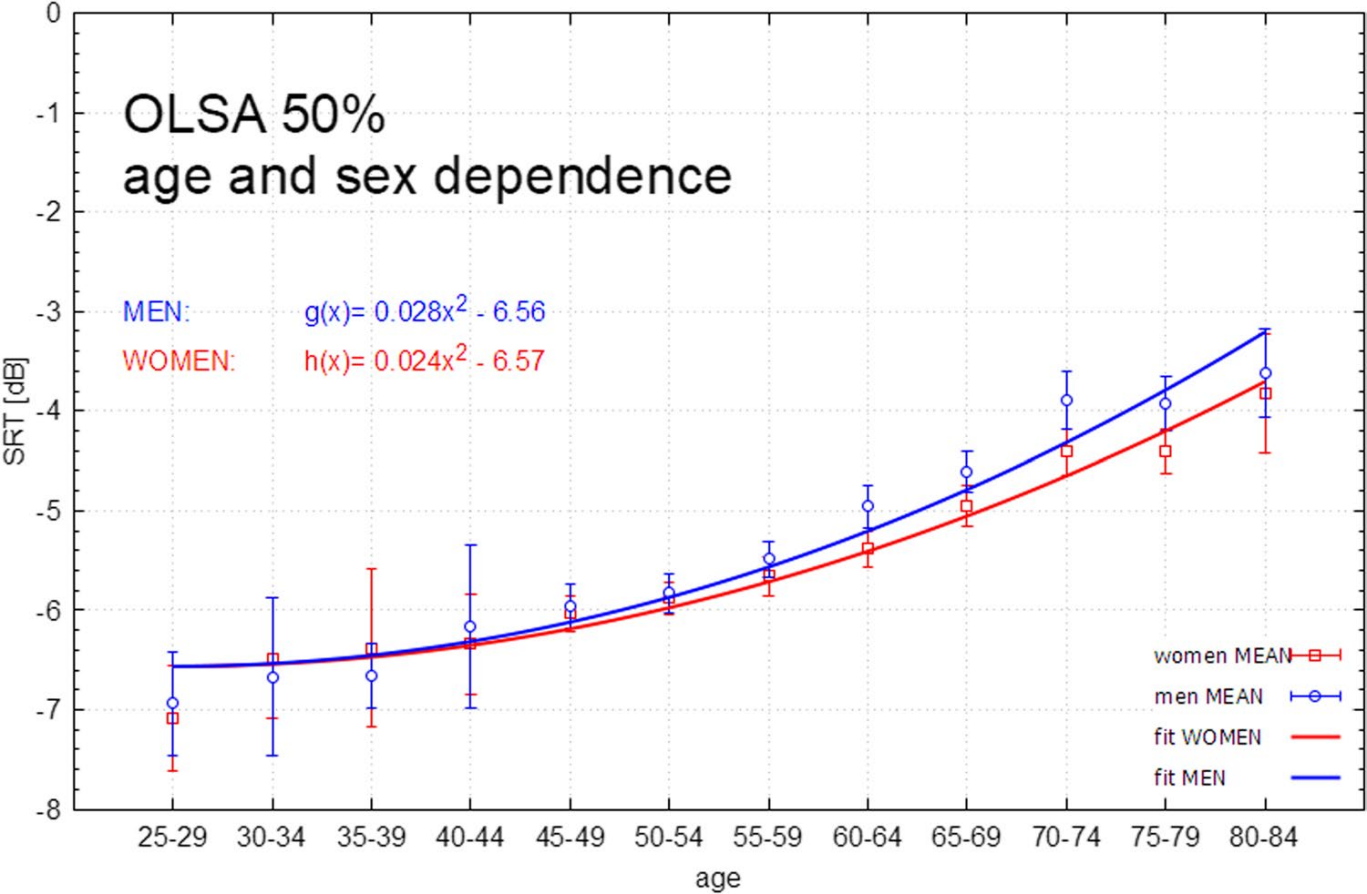
OLSA SRT (men and women) stratified by age decades (5-year intervals) in the entire study cohort (including hearing disorders, n = 2900 participants). Error bars include interquartile ranges. *OLSA* Oldenburg Sentence Test (German Matrix Test), *dB* decibel, *SRT* speech recognition threshold, % percent, fit error men [g(x) = ax^2^ + b]: **a** 10.2%, **b** 1.8%, fit error women [h(x) = ax^2^ + b] **a** 3.5%, **b** 0.5%

**Fig. 3:**
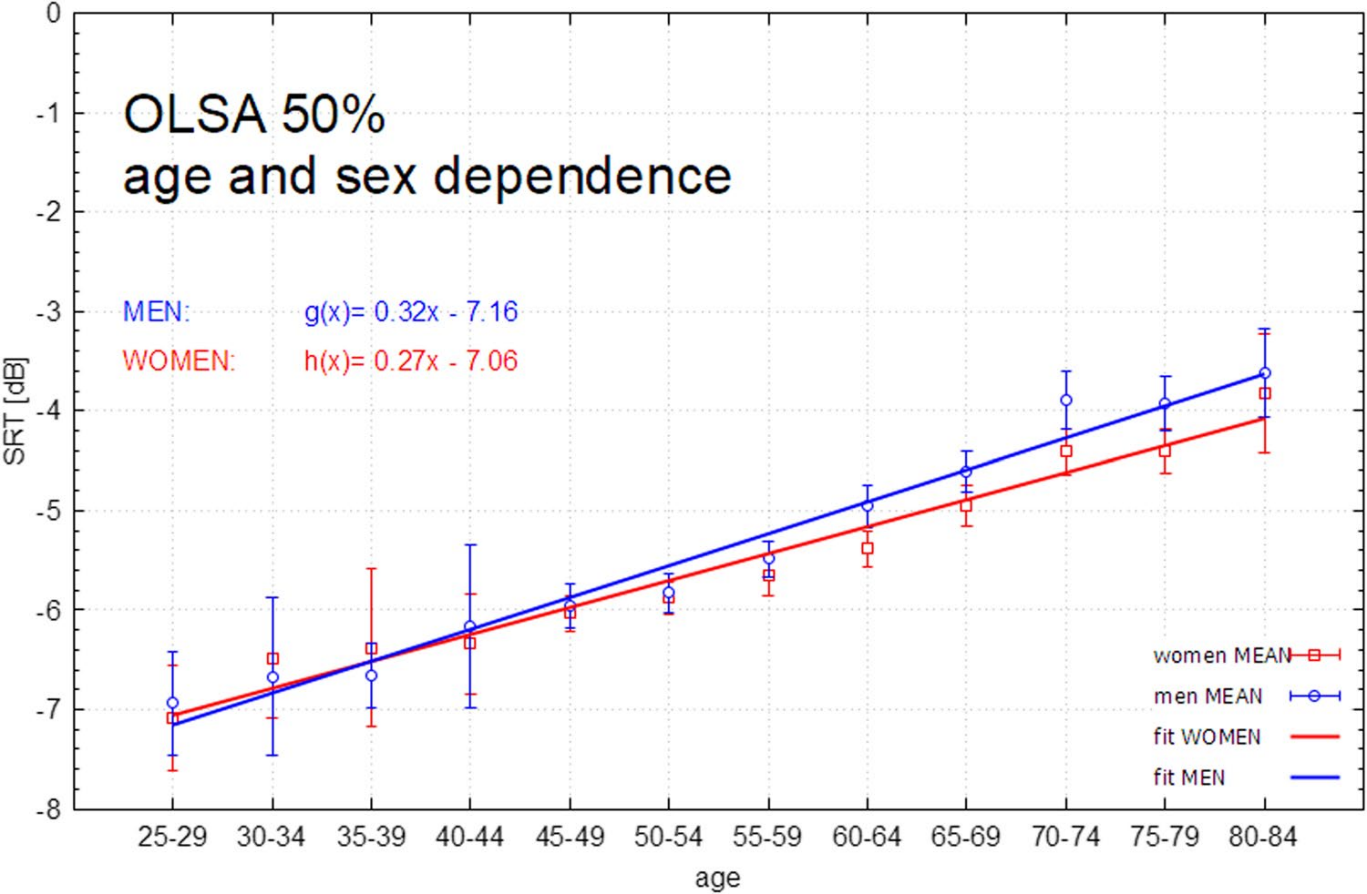
OLSA SRT (males and females) stratified by age decades (5-year intervals) without hearing loss (main cohort participants with a hearing loss of > 20 dB at frequencies 0.5/1/2/4 kHz excluded, n = 1258 participants, linear fit model). Error bars show interquartile ranges. *OLSA *Oldenburg Sentence Test (German Matrix Test), *dB *decibel, *SRT *speech recognition threshold, fit error men [g(x) = ax + b]: **a** 7.7%, **b** 1.7%, fit error women [h(x) = ax + b] **a** 6.6%, **b** 1.4%

**Fig. 4:**
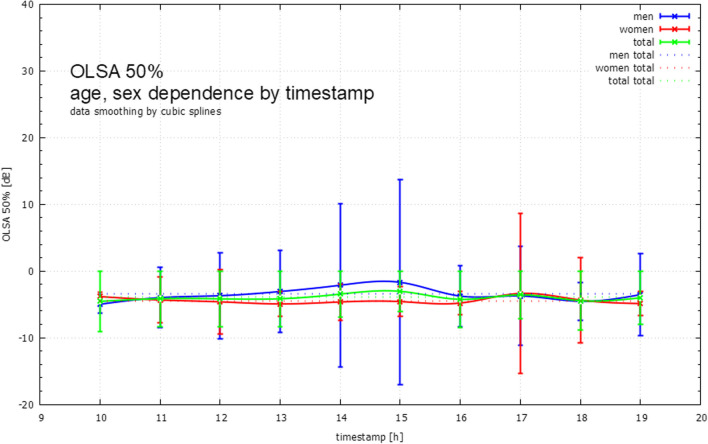
OLSA SRT (men and women) stratified by time-stamps (1-h intervals). The figure shows the mean performance in the OLSA performed at different times of the day. Error bars show interquartile ranges. *OLSA *Oldenburg Sentence Test (German Matrix Test) *dB *decibel, *SRT *speech recognition threshold, *h *hours

The results are plotted in Figs. 2, 3, 4. Figure 2 shows male and female participants plotted separately in the age cohorts and the OLSA 50% SRT values achieved in each cohort. Figure 3 shows the same order of presentation without the participants with a hearing loss of > 20 dB. (0.5, 1, 2, 4 kHz were included). Figure 4 shows the SRT of the OLSA plotted by time of the day and sex.

The overall SRT for the OLSA in the main cohort was − 3.5 ± 7.7 dB for male subjects and − 4.5 ± 5.6 dB for female subjects (male and female combined: − 4.0 ± 6. 8 dB).

The SRT for the OLSA showed a mean for the youngest group of − 6.9 ± 1.0 dB (Group 1 male) and − 7.1 ± 0.8 dB (Group 1 female) with a steady increase over the course of the age cohorts. The overall results in group 2 (30–34 years of age, male and female combined) show an OLSA SRT of − 6.6 ± 1.2 dB, in group 3 (35–39 years of age) − 6.5 ± 1.3 dB, in group 4 (40–44 years of age) − 6.0 ± 1.7 dB, group 5 − 5.9 ± 1.2 dB, group 6 − 5.8 ± 1.3 dB, group 7 − 5.4 ± 1.5 dB, in group 8 (60–64 years of age) − 4.9 ± 3.6 dB, group 9 − 4.1 ± 3.3 dB, group 10 − 2.9 ± 7.7 dB, group 11 − 2.0 ± 7.6 dB. There is one exception to the trend of the steady increase: Group 12 (80–84-year old participants), both male and female, show a positive SRT (male: 2.8 ± 16.8 dB; female: 1.7 ± 19.2 dB) (n = 205 total). Complete results are shown in Table 1.

**Table 1:** Mean results of OLSA SNR in dB in relation to the age groups and intragroup standard deviations

Group	1	2	3	4	5	6	7	8	9	10	11	12	13	
Age decade (years)	25–29 n = 26	30–34 n = 30	35–39 n = 42	40–44 n = 30	45–49 n = 254	50–54 n = 364	55–59 n = 406	60–64 n = 357	65–69 n = 428	70–74 n = 345	75–79 n = 401	80–84 n = 205	85–89 n = 12	Total
Men (in dB)	− 6.9 ± 1.0	− 6.7 ± 1.3	− 6.7 ± 0.7	− 6.2 ± 1.1	− 5.9 ± 1.3	− 5.7 ± 1.3	− 5.3 ± 1.7	− 4.4 ± 4.9	− 3.9 ± 3.0	− 2.1 ± 10.1	− 1.1 ± 9.5	2.8 ± 16.8	− 0.7 ± 3.6	− 3.5 ± 7.7
Women (in dB)	− 7.1 ± 0.8	− 6.5 ± 1.2	− 6.4 ± 1.7	− 6.0 ± 1.0	− 5.9 ± 1.2	− 5.9 ± 1.24	− 5.6 ± 1.3	− 5.3 ± 1.2	− 4.3 ± 3.7	− 3.6 ± 3.5	− 3.1 ± 4.4	1.7 ± 19.2	− 2.5 ± 2.1	− 4.5 ± 5.6
Total (in dB)	− 7.00 ± 0.9	− 6.6 ± 1.2	− 6.5 ± 1.3	− 6.04 ± 1.70	− 5.9 ± 1.2	− 5.8 ± 1.3	− 5.4 ± 1.5	− 4.9 ± 3.6	− 4.1 ± 3.3	− 2.9 ± 7.7	− 2.0 ± 7.6	2.4 ± 17.8	− 1.5 ± 3.1	− 4.0 ± 6.8

*OLSA* Oldenburg Sentence Test (German Matrix Test), *dB* decibel, *SNR* signal–noise-ratio, *n* number of participants

As a linear relationship between SRT in the OLSA and age decade seems doubtful, a quadratic relationship was used for further analysis. The assumption of a simple quadratic relationship by means of a parabolic equation (g = g(x) = a∙x^2^ + b) as opposed to a linear function (y = f(x) = m∙x + n) as seen in Fig. 2 (combined dependence male and female) results in a better fit. A left shift of the parabolic function (g = g(x) = a∙x^2^ + bx + c) was implemented in the fit. The influence of “b” was found to be negligible. In order to have as few degrees of freedom as possible, the degree of freedom with the least influence was omitted.

As a sensitivity analysis, any participant with hearing loss was removed from the total cohort in Table 2 to examine if there is an influence of the physiological aging in the normal hearing population on the OLSA outcome. Thus, Table 2 shows only participants with a hearing threshold < 20 dB (normal hearing). As a result of this approach, 1642 participants were excluded from the main cohort. Therefore, 1258 participants remained in this subcohort. Note that the decade 85–89 does not consist of any participants without hearing loss. The group of the 80–84-year-old had only five participants with no hearing loss (4 females and 1 male), so no significant values can be reported for the group. This is why, we switched to decade groups for further analysis.

**Table 2:** Mean results of OLSA SRT in dB in relation to the age groups (sample without hearing impairment < 20 dB of 0,5/1/2/4 kHz)

Group	1	2	3	4	5	6	7	8	9	10	11	12	13	
Age decade (years)	25–29 n = 25	30–34 n = 29	35–39 n = 37	40–44 n = 28	45–49 n = 195	50–54 n = 282	55–59 n = 261	60–64 n = 167	65–69 n = 131	70–74 n = 60	75–79 n = 38	80–84 n = 5	85–89 n = 0	Total
Men (in dB)	− 6.9 ± 1.0	− 6.7 ± 1.4	− 6.7 ± 0.7	− 6.3 ± 1.1	− 6.0 ± 1.3	− 6.0 ± 1.1	− 5.7 ± 1.0	− 5.2 ± 1.2	− 5.0 ± 1.2	− 4.4 ± 1.4	− 4.7 ± 1.1	− 4.0 ± NaN	NaN	− 5.7 ± 1.3
Women (in dB)	− 7.3 ± 0.4	− 6.5 ± 1.2	− 6.3 ± 1.8	− 6.3 ± 1.0	− 6.1 ± 1.0	− 6.0 ± 1.1	− 5.8 ± 1.1	− 5.6 ± 1.1	− 5.2 ± 1.2	− 4.8 ± 1.1	− 4.6 ± 0.9	− 5.2 ± 0.5	NaN	− 5.8 ± 1.2
Total (in dB)	− 7.1 ± 0.8	− 6.6 ± 1.3	− 6.5 ± 1.3	− 6.3 ± 1.1	− 6.1 ± 1.1	− 6.0 ± 1.1	− 5.7 ± 1.1	− 5.4 ± 1.1	− 5.1 ± 1.2	− 4.6 ± 1.2	− 4.6 ± 1.0	− 5.0 ± 0.7	NaN	− 5.7 ± 1.2

*OLSA* Oldenburg Sentence Test (German Matrix Test), *dB* decibel, SNR signal–noise-ratio. *kHz* kilohertz, *NaN* not a number, *n* number of participants

Table 2 shows the SRT for the German Matrix Test (OLSA) for participants classified by age group without hearing loss > 20 dB at the frequencies 0.5/1/2/4 kHz. The overall mean in this subcohort for male and female participants is − 5.7 ± 1.2 dB. The OLSA SRT shows a linear increase over the course of the age cohorts without hearing loss > 20 dB, congruent with the OLSA SRT in the main cohort. A linear regression model adjusted for age shows a high statistical and thus a linear relationship between OLSA SRT and age.

We hypothesized that the higher variability of SRT with increasing hearing loss might be the cause of a higher variability in SRT across age, if hearing loss is the intermediate factor. This is why the variance was tested, too. When comparing the variance of the different age decades using Levene's test, it yielded significant results, which means that the variances between the age decades do not appear to be equal. Therefore, we performed the Levene test only on participants aged < 60 years. It was found that the variance between age decades was not significantly different (p = 0.142) (see Appendix 1). However, the repetition of a specific ANOVA for cases where the assumption of equal variances between the age groups is violated (the Welch ANOVA) still yields a significant result for age (Appendix 1).

In order to test the effect of age on the OLSA by comparing the results for each age decade to the results from the youngest decade (25–34 years old), the Dunnett-test was performed. We checked whether the parameters in the models differ between age decades. This was found to be true (see Appendix 1). The age groups over 60 were significantly different from the reference group (youngest decade).

To ensure that the observed age dependence was a direct effect of aging and not an indirect effect mediated by increased hearing loss with age, we performed an analysis of variance (ANOVA) testing the interaction of age × hearing loss on OLSA scores (dependent variable), only for participants with normal hearing (< 20 dB). While the interaction age × hearing loss was significant with a p value of < 0.001 for the entire sample (including those with hearing loss), the same interaction was not significant for the participants without hearing loss (only < 20 dB) with a p value of 0.193 (see Appendix 1, first and third model ANOVA). This provides clear evidence that the observed age dependence of the SRT is independent of the increase in SRT caused by a hearing loss.

The results regarding the time-of-day dependency can be derived from Table 3 and are shown graphically in Fig. 4. The data are presented as (x, y) point clouds separated by sex. Cubic splines (Fig. 4) are computed and plotted as compensation curves. In general, the SRT OLSA values of men and women appear to be different and variable throughout the day. Interestingly, the results for men and women are very close to each other between 10:30 h–11:30 h and 17:00 h–1800 h, but drift apart at noon. It was not possible to reach statistical significance for the time of day dependency when performing the German Matrix Test

**Table 3:** Mean results of OLSA SNR in dB in relation to the time-of-day and intragroup standard deviation

Timestamp	1000-1100h	1100-1200 h	1200–1300h	1300–1400 h	1400–1500 h	1500–1600 h	1600–1700 h	1700–1800 h	1800–1900 h	1900–2000 h	Total
Men (in dB)	− 5.0 ± 1.3	− 4.0 ± 4.5	− 3.7 ± 6.5	− 3.1 ± 6.1	− 2.1 ± 12.2	− 1.7 ± 15.4	− 3.8 ± 4.6	− 3.7 ± 7.4	− 4.5 ± 2.8	− 3.5 ± 6.2	− 3.5 ± 7.7
Women (in dB)	− 3.8 ± 0.6	− 4.3 ± 3.5	− 4.6 ± 4.9	− 4.9 ± 1.8	− 4.6 ± 2.7	− 4.6 ± 2.3	− 4.8 ± 1.7	− 3.4 ± 12.0	− 4.4 ± 6.4	− 4.9 ± 1.8	− 4.5 ± 5.6
Total (in dB)	− 4.6 ± 1.2	− 4.2 ± 4.1	− 4.2 ± 5.7	− 4.2 ± 4.3	− 3.5 ± 8.7	− 3.0 ± 11.4	− 4.3 ± 3.5	− 3.6 ± 9.7	− 4.5 ± 4.9	− 4.0 ± 5.1	− 4.0 ± 6.8

*OLSA* Oldenburg Sentence Test (German Matrix Test), *dB* decibel, *SNR* signal–noise-ratio, *h* hours

## Discussion

In this study, we established an age standardization for the OLSA in adults representing the general population of the city of Mainz and its district of Mainz-Bingen, Germany. We found that with increasing age of the participants, there is a decrease in performance on the German Matrix Test [Oldenburg Sentence Test (OLSA)]. By excluding participants with hearing impairment, we can postulate a meaningful age standardization for the OLSA in the adult population with normal hearing.

The strength of this study lies in the clinical rigor of testing all participants with pure-tone audiometry in a soundproof booth, the pure number of participants for the OLSA, and the standards of the University Department of Otolaryngology. This design provides representative audiometric data from the largest adult cohort in Germany known to the authors to date.

This study cohort consists of citizens from a combination of urban and rural areas, although the city and county are geographically adjacent. We do not expect a difference between urban and rural participants, as both are located in a highly industrialized and at the same time densely populated region. Due to its central location, the region is assumed to be representative of the German population [8].

Difficulty understanding speech in situations with some background noise (“cocktail party phenomenon”) is the most common complaint of patients with sensorineural hearing loss. The ability to understand spoken sentences in noise is poorly predicted by pure-tone-thresholds alone [18]. Functional speech-in-noise tests have been developed to assess this type of hearing loss [18].

The reference values for the OLSA (in adults) are given as − 7.1 dB SRT with an increase of 17.1% pp (percentage points)/dB of the absolute speech understanding score/signal-to-noise ratio change of 1dB [7, 15]. To the authors’ knowledge, no study of the magnitude of the present study has been conducted. This study presents a total number of 2900 documented OLSAs. Due to the large number of OLSAs and the data collected in the age groups, we propose this age standardization for the OLSA. As can be seen in the results section, there is a continuous decrease in the 50% SRT of the OLSA with increasing age of the participants. The exception in the age group of 80–84 years can be explained by the very high incidence of hearing losses in this group. After removing the participants with hearing impairment (≥ 20 dB), only five participants with normal hearing remained in the 80–84 age group. Thus, the prevalence of hearing impairment in older patients is therefore probably too high to postulate an age standardization in our study population. It is therefore possible that an age standardization for the OLSA could be evaluated up to an age limit of 80 years.

In contrast to the OLSA, the Oldenburg Children's Sentence Test (OLKISA) is used with an age standardization to allow for an adjustment of the scoring. Weissgerber et al. showed that the OLKISA can be used to assess speech perception with comparable accuracy to adults, with the advantage of a higher sensitivity compared to single word tests [14].

Another speech audiometric test is the Freiburg Speech Test. It consists of a numerical test and a monosyllabic test. It is easy and quick to perform and is the most commonly used speech test in Germany [19]. The Göttinger Sentence Test is less time-consuming than the OLSA, but has a high risk for list redundancy when repeatedly tested. Only 20 test lists with 10 sentences each are available [20]. Because of the many variable test lists, the OLSA can be administered as often as desired to the same subject. The Göttinger Sentence Test and the Freiburg Speech Test are not age-standardized. Due to the limited number of test lists in the Göttinger Sentence Test and the lack of complex sentences in the Freiburg Speech Test, the OLSA seems to be more clinically relevant.

Clinical data and modelling work show that the SRT (measured with the German Matrix Test) increases with increasing average hearing loss (approximately < 1 dB SRT loss per 10 dB hearing loss- independent of age [21, 22]. By eliminating the factor of hearing loss in an ageing society (by eliminating hearing loss > 20 dB) a single age-dependent factor can be demonstrated (also see Appendix 1 for full statistical analysis).

The variances in the age groups < 60 years were not significantly different according to Levene’s test. The OLSA appears to be stable for interindividual variance in these groups. However, the older age groups have a different variance than the younger groups. The increased variance in the older age groups may be related to a higher prevalence of hearing loss in these groups. The Welch ANOVA was performed for this case and still yielded significant results for age. This suggests that the OLSA performance is independent of hearing loss in all the age groups.

The results for the diurnal dependence could not be shown to be statistically significant due to the wide range of standard errors. Therefore, only a descriptive statistic is displayed.

In general, the OLSA SRT values of men and women appear different and variable during the course of the day. Interestingly, the results for men and women are very close between 10:30 am and 11:30 am and between 5:00 pm and 6:00 pm, while they diverge significantly at noon. Since the OLSA is generally a test procedure that requires a certain degree of concentration and intelligence, concentration problems (e.g. lunchtime) of the subjects as well as uncertainties in the test execution could be a possible cause. Regarding the diurnal dependence, potential confounders (age, sex, and potential hearing impairment) must be considered.

This study has several limitations, which are discussed below. First, the GHS is designed as a population-based cohort study and by its design is representative for the population of Mainz (city) and Mainz-Bingen (county), Germany [15]. Otologic examination was included in the study and performed for the first time at the 10-FU examination. Approximateley 20% of the original baseline cohort (10,000 participants) were lost to follow-up due to mortality, refusal to participate again or migration. This results in a certain selection bias of a not presentable dimension. In addition, a further 2759 participants were excluded due to missing otologic examination data. This was generally due to absence of study staff. We assume that this is a random phenomenon.

Another exclusion criterion for the GHS study was physical and mental disability. The prevalence of hearing loss is higher in people with comorbidities such as diabetes or cardiovascular disease [23]. Excluding these participants from a study could lead to an underestimation of the prevalence of hearing loss and therefore the performance in OLSA. However, it should be noted, that this only applies to the total cohort. The fact that subjects with hearing loss were removed from the subcohort minimized this variable. However, we cannot completely rule out the possibility that some residual participants with subthreshold or high frequency hearing loss are included, as they may have slipped through our > 20 dB criterion and thus have still remained in the subgroup (see below).

In our sample without hearing loss, group 12 consists of only five participants, as the majority of the original group in the full cohort (n = 205) had to be excluded from the subcohort due to hearing loss. The authors would like to emphasize the increasing prevalence of hearing loss in the aging population [24] and the need to screen for hearing impairment in older adults.

Considering the aging of the population, there is a significant population over 87 years of age. In our sample, 100% of the participants were under the age of 89 years. Removing this last age category of 85+ gives a more unbiased result that only considers those under 85 years of age. Thus, the proposed age standardization can only be accurately applied to adults under the age of 85. Our subcohort (adults without hearing loss) over the age of 80 has only 5 participants.

It cannot be completely ruled out that even after removing the subgroup of > 20 dB, there are still group members remaining with an undetected hearing loss. This is especially true for the group from 40+ age group. In everyday life, we often see sloping curves in the frequencies from 3–4 kHz that increase with age. The question remains as to how someone with a mean audiometric hearing loss of < 20 dB up to 4 kHz, but with progressively decreasing thresholds > 4 kHz, would perform in the OLSA. This uncertainty still prevents a clear standardisation of OLSA across age.

## Conclusion

Our study showed a clear age dependence of OLSA. A study with this number of evaluable Oldenburg Sentence Tests is a novelty and the results show a representative population of the population in Mainz and surroundings. By eliminating the age dependence of audiometric hearing loss by including only normal hearing listeners, we postulate an age-standardized scale (Fig. 3) for the assessment of OLSA in the adult population. Understanding this age dependence will be the basis for further understanding of OLSA as well as audiological understanding in the general population. With some limitations regarding subthreshold and high frequency hearing loss kept in mind, it will be possible to correctly evaluate and use the OLSA results according to age, especially with regard to hearing aids as well as hearing aid provision and fitting. In the future, it would be interesting to study OLSA performance in relation to cognitive decline to determine if the OLSA is able to detect cognitive deficits.

Time of day performance on the OLSA shows interesting gender differences, although no statistical significance could be shown. Further studies to identify possible confounders should be initiated.

## Appendix 1: Performed statistical tests:

ANOVA: Analysis of Variance, testing the contribution of hearing impairment and age to performance in the OLSA.

Levene Test: Testing the assumption of homoscedasticity, homogenity of variance.

Dunnett Test: Testing differences in variance in relation to youngest age decade as control.

Analyses of Variance (ANOVA) for interaction between age and hearing loss in relation to the results of the OLSA for the whole sample, Df = degrees of freedom,

**Table:**

	Df	F-value	p-value
Age	1	282.7	< 0.0001
Hearing loss	1	55.6	< 0.0001
Hearing loss × age	1	53.8	< 0.0001

(a) First model (ANOVA): Dependent variable: OLSA Results, independent variable: age decades (10y) age p-value < 0.0001-significant, interaction age*hearing impairment p-value < 0.0001-significant.

**Table:**

	Df	F-value	p-value
Age	1	332.2	< 0.0001
Hearing impairment (cont.)	1	521.8	< 0.0001
Hearing impairment*age	1	136.5	< 0.0001

First model Levene test: p value < 0.0001, variances do not seem to be equal between age decades.

(b) Second Model (ANOVA): Dependent variable: OLSA Results, independent variable: age decades only < 60 y (10 y), age p-value < 0.0001-significant, interaction age*hearing impairment p-value 0.3-non-significant.

**Table:**

	Df	F-value	p-value				
Age	1	85	< 0.0001				
Hearing impairment (cont.)	1	133	< 0.0001				
Hearing impairment*Age	1	1.1	0.3				

Second model Levene Test: p value 0.1423, variances seem to be equal in the younger age decades.

Additional model (Welch-ANOVA): p value < 0.0001.

Dunnett test

Dependent variable: OLSA Results, independent variable: age decades (10 y), reference: 25–34 old persons.

p-value comparison 35–44 years with 25–34 years: 0.95860.

p-value comparison 45–54 years with 25–34 years: 0.55503.

p-value comparison 55–64 years with 25–34 years: 0.16035.

p-value comparison 65–74 years with 25–34 years: 0.00085.

p-value comparison 75–89 with 25–34: < 0.0001.

Parameters appear to differ in oldest decade compared to youngest decade.

(c) Third Model (ANOVA): Dependent variable: OLSA Results, independent variable: age decades, sample: only non-hearing impaired individuals (< 20 dB hearing impairment: age p-value < 0.0001—significant interaction, interaction age × hearing impairment p-value 0.193—non-significant interaction.

**Table:**

	Df	F value	Pr(> F)
Age	1	371.7	< 0.0001
Hearing impairment (cont.)	1	18.1	< 0.0001
Hearing impairment × age	1	1.7	0.193
